# Interaction of Glucagon G-Protein Coupled Receptor with Known Natural Antidiabetic Compounds: Multiscoring *In Silico* Approach

**DOI:** 10.1155/2015/497253

**Published:** 2015-07-06

**Authors:** M. H. Baig, K. Ahmad, Q. Hasan, M. K. A. Khan, N. S. Rao, M. A. Kamal, I. Choi

**Affiliations:** ^1^School of Biotechnology, Yeungnam University, Gyeongsan 712749, Republic of Korea; ^2^Department of Biosciences, Integral University, Lucknow 226026, India; ^3^Department of Bioengineering, Integral University, Lucknow 226026, India; ^4^School of Computational and Integrative Sciences, Jawaharlal Nehru University, New Delhi 110067, India; ^5^King Fahd Medical Research Center, King Abdulaziz University, P.O. Box 80216, Jeddah 21589, Saudi Arabia; ^6^Enzymoics, 7 Peterlee Place, Hebersham, NSW 2770, Australia

## Abstract

Glucagon receptor (GCGR) is a secretin-like (class B) family of G-protein coupled receptors (GPCRs) in humans that plays an important role in elevating the glucose concentration in blood and has thus become one of the promising therapeutic targets for treatment of type 2 diabetes mellitus. GCGR based inhibitors for the treatment of type 2 diabetes are either glucagon neutralizers or small molecular antagonists. Management of diabetes without any side effects is still a challenge to the medical system, and the search for a new and effective natural GCGR antagonist is an important area for the treatment of type 2 diabetes. In the present study, a number of natural compounds containing antidiabetic properties were selected from the literature and their binding potential against GCGR was determined using molecular docking and other* in silico* approaches. Among all selected natural compounds, curcumin was found to be the most effective compound against GCGR followed by amorfrutin 1 and 4-hydroxyderricin. These compounds were rescored to confirm the accuracy of binding using another scoring function (*x*-score). The final conclusions were drawn based on the results obtained from the GOLD and *x*-score. Further experiments were conducted to identify the atomic level interactions of selected compounds with GCGR.

## 1. Introduction

Diabetes mellitus is a group of metabolic diseases in which the human body is unable to utilize and store available glucose, which results in the blood glucose level rising above the threshold level. Globally, diabetes has affected 347 million people to date [1]. The most common symptoms of this disease include weight loss, polyuria, polydipsia, and polyphagia [2]. Whenever glucose levels decrease in the blood (such as under fasting situations), glucagon-a 29-amino acid peptidal hormone is secreted by pancreatic *α*-cells, which enhances the blood glucose level [3]. Increased glucagon in the blood leads to the promotion of glycogenolysis and gluconeogenesis in the liver, while the insulin inhibitory effect of these processes is attenuated, ultimately enhancing the blood glucose level [4]. The combined action of insulin and glucagon is required to maintain glucose homeostasis inside the body [5, 6]. Therefore, two strategies have been applied to control diabetic hyperglycemia to date, reducing circulating glucagon levels and inhibiting glucagon mediated effects in target body tissues and cells. Several studies have demonstrated significant blood glucose lowering effects in diabetic animal models through application of potent peptide antagonists [7, 8], and immunoneutralization of glucagon in diabetic animals has been shown to reduce glucagon-stimulated hyperglycemia [9, 10].

The glucagon receptor (GCGR) is a 62 kDa protein activated by glucagon that is a member of G-protein coupled receptor (GPCR) superfamily [11]. In humans, the glucagon receptor is encoded by the* GCGR* gene [12, 13]. Glucagon receptors are primarily expressed in the liver and kidney, with lesser amounts found in the heart, adipose tissue, spleen, thymus, adrenal glands, pancreas, cerebral cortex, and gastrointestinal tract. By binding to GCGR, glucagon sends a signal inside the cell, which activates adenylyl cyclase, leading to the generation of high cAMP levels [14]. In addition, GCGR also couples to an intracellular Ca^2+^-mediated pathway [15]. GCGR activation leads to increase in metabolic processes such as glycogenolysis and gluconeogenesis, resulting in increased glucose concentrations in hepatic cells and tissues [16, 17].

Since GCGR plays an important role in elevating the glucose concentration in blood (glycemia) and there are many small-molecule inhibitors available for receptors of the GPCR family [18], it is a potent target for the development of small-molecule antagonist/inhibitors. A number of antagonists with varying degrees of potency and structures have been reported in recent years [19]. GCGR based inhibitors for the treatment of type 2 diabetes are either glucagon neutralizing antibodies [20, 21] or small molecular antagonists [22–24]. These compounds have been shown to effectively terminate the GCGR action. However, concerns about safety, tolerance, and stimulation of adverse immune response when using these types of agents against GCGR for the treatment of type 2 diabetes have led to investigations to identify drugs or compounds of natural origin to combat this problem. Indeed, GCGR antagonist/inhibitors of natural origin may be safe and favorable therapeutic agents for the treatment of type 2 diabetes. Accordingly, it is important to search for new and effective GCGR antagonists from natural sources [25]. Therefore, the present study was conducted to search for natural antagonists against GCGR* in silico*. All natural compounds selected in this study were collected from the available literature and have been reported to have antidiabetic properties [26–28]. Additionally, molecular docking studies have been conducted to investigate the binding affinity of all selected compounds. The results were then reevaluated using two different scoring functions to confirm the accuracy of our results. Overall, the results presented herein enabled calculation of the accessible surface area (ASA) of GCGR (uncomplexed) and its docked complex with the selected compounds for analysis of quantification of the packing of residues in GCGR before and after the binding of ligands.

## 2. Methods

### 2.1. Preparation of Enzyme and Ligand for Docking

The 3D crystal structure of human GCGR was retrieved from the RCSB protein databank (pdb ID: 4L6R) (http://www.rcsb.org/pdb/explore/explore.do?structureId=4L6R) [29]. Before conducting the molecular docking calculations, all water molecules and other heteroatoms were removed. A CharMm [30] force field was applied to the structure of GCGR, followed by 1000-step energy minimization using the steepest descent method. The Distance-Dependent Dielectrics type implicit solvent model was used to conduct the energy minimization step with the RMS gradient set to 0.1. A total of 83 natural compounds were selected in this study. All natural compounds used in this study were selected from the available literature. The 3D structures of all natural compounds were extracted from the PubChem Compound database. A Cff force field [31], which is a general purpose class II force field with good parameter coverage for many organic molecules, was applied to all the structures. As class II force field, it has additional cross terms in its potential energy function relative to other class I force fields.

### 2.2. Molecular Docking

Molecular docking was conducted to investigate the interaction of all the natural compounds against GCGR. Genetic Optimization for Ligand Docking 5.0 (GOLD) [32] was used for docking of all the selected compounds against GCGR. The annealing parameters were set to 5.0 and 2.5 to evaluate van der Waals and hydrogen bonding docking, respectively. The population size was set to 100 with a selection pressure of 1.2. The number of operations was fixed to 1,00,000, with 5 islands, a niche size of 2, migration value of 10, mutation value of 100, and crossover of 100. The binding energies of docked molecules were also calculated using *x*-score [33]. All molecular graphics material of docked complexes was prepared using Pymol.

### 2.3. Accessible Surface Area Calculation

Differences in the accessible surface area (ASA) of the GCGR before and after the binding of identified inhibitors were calculated for validation of effectiveness of these compounds using NACCESS version 2.1.1 [34]. The accessible surface area, *A*, of an atom is the area on the surface of a sphere of radius *R*, on each point of which the center of a solvent molecule can be placed in contact with this atom without penetrating any other atom of the molecule. The radius *R* is given by the sum of the van der Waals' radius of the atom and the selected radius of the solvent molecule. An approximation to this area is computed by this program using the following formula.

Accessible surface area is

(1) $$\begin{array}{c} A=\sum \left(\;\frac{R}{\sqrt{R^{2}-Z_{i}^{2}}}\;\right)\cdot D\cdot L_{i},\;D=\frac{\Delta Z}{2}+\Delta^{\prime }Z, \end{array}$$

where *L*_*i*_ is the length of the arc drawn on a given section *i*, *Z*_*i*_ is the perpendicular distance from the center of the sphere to the section *i*, Δ*Z* is the spacing between the sections, and Δ′*Z* is Δ*Z*/2 or *R* − *Z*_*i*_, whichever is smaller. Summation is over all of the arcs drawn for the given atom. The accessibility is defined simply as the accessible surface area divided by 4*πR* ^2^ multiplied by 100.

## 3. Results and Discussion

**Figure 1:**
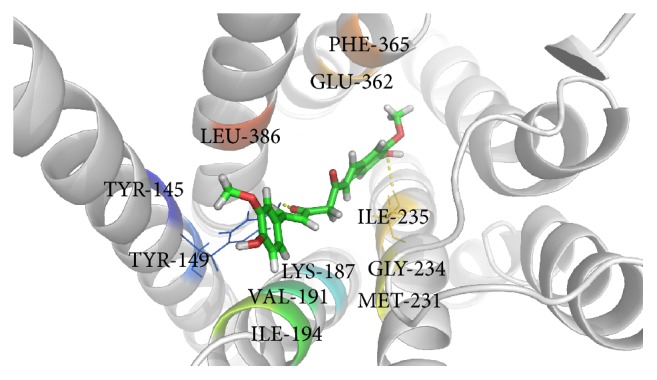
Binding of curcumin within the active site of GCGR.

**Figure 2:**
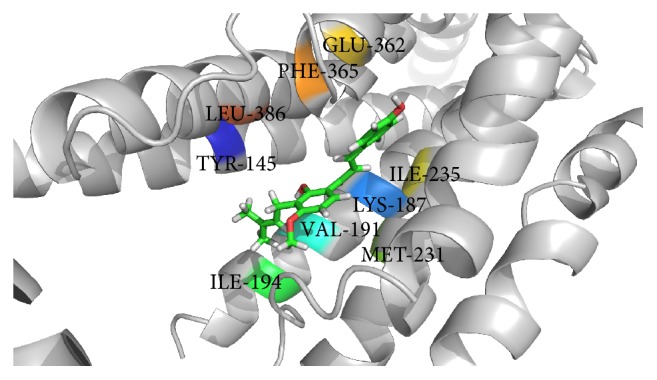
Binding of 4-hydroxyderricin within the active site of GCGR.

**Figure 3:**
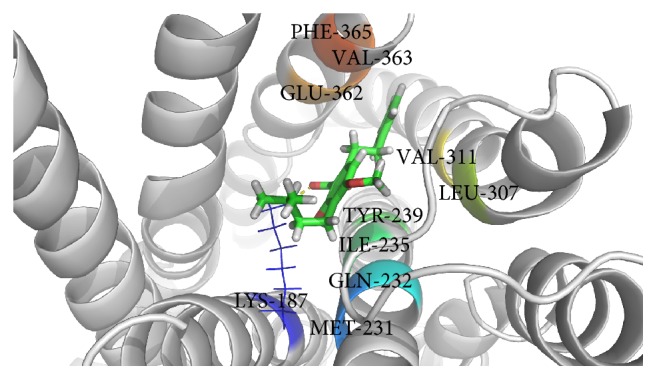
Binding of amorfrutin 1 within the active site of GCGR.

**Figure 4:**
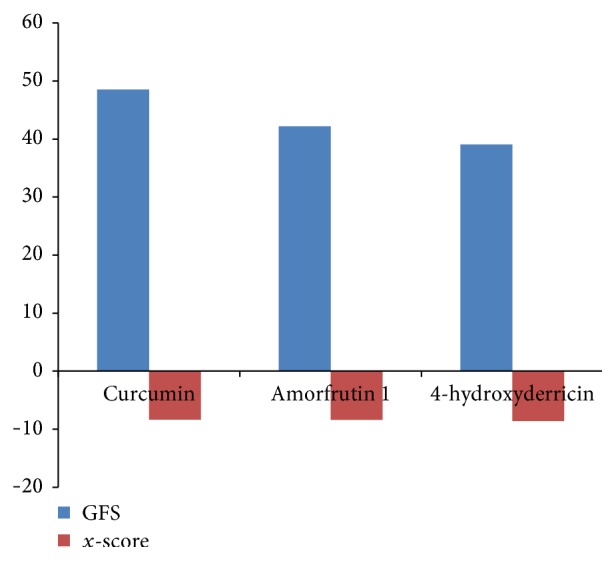
Comparison of both scoring functions used in this study.

Glucagon G-protein coupled receptor, class B GPCR, has become a promising therapeutic drug target for the treatment of type 2 diabetes mellitus (T2DM) [35, 36]. Earlier studies have reported that blockade of glucagon receptor gene (GCGR) activity is useful for the treatment of T2DM [25, 35, 36]. However, management of diabetes without any side effects is still a challenge to the medical system [37]. This has led to increasing demand for natural products with antidiabetic activity with fewer or no side effects. Molecular docking is considered to be an important tool for investigation of the mode of interaction of ligands with the target and elucidation of the underlying binding mechanism [38, 39]. In this study, we determined the binding potential of several natural compounds with known antidiabetic properties against GCGR using molecular docking and other* in silico* approaches. The prime objective of the present study was to identify the binding potential of several natural antidiabetic compounds against GCGR using the molecular docking approach. In this regard, we used an* in silico* approach to identify natural compounds with the potential for use in the treatment of GCGR. Additionally, molecular docking simulation studies were conducted to investigate possible binding modes of all selected natural compounds against GCGR. Several plausible binding modes were detected and ranked based on their gold fitness score. Moreover, these compounds were rescored to confirm the accuracy of binding using another scoring function (*x*-score). The final conclusions were drawn based on the results obtained from GOLD and the *x*-score. Curcumin, a principal component of turmeric (*Curcuma longa *Linn.) and a popular spice in Asian cuisine, was found to be the most effective against GCGR (gold fitness score of 53.53), followed by amorfrutin 1, widely available traditional medicine isolated from licorice (*Glycyrrhiza foetida*), and 4-hydroxyderricin, isolated from root of* A. keiskei*, which were found to bind with gold fitness scores of 48.18 and 44.06, respectively. Rescoring of these docked results using *x*-score revealed that curcumin, amorfrutin 1, and 4-hydroxyderricin bind within the active site of GCGR with binding free energies of −8.35, −8.37, and −8.56 kcal/mol, respectively.  Table 1 illustrates the binding score of the finally selected compounds against GCGR. The binding mode of the selected inhibitors within the active site of GCGR is shown in Figures 1–3. The results obtained from both scoring functions were also found to be in good agreement with each other. The scores obtained using all three functions are shown in the graph (Figure 4).

This study revealed that the binding of all natural compounds within the active site of GCGR is largely dominated by hydrophobic interactions. There were only three amino acid residues of GCGR (K187, Y149, and I235) found to participate in generation of hydrogen bonds with curcumin and amorfrutin 1. V191, I194, M231, I235, E362, and F365 were found to be common active site residues involved in the proper accommodation of natural compounds within the active site of GCGR via hydrophobic contact. The role of these important active site residues has already been discussed in previous studies [25, 40]. Further experiments were conducted to identify atomic level interactions of finally selected compounds with GCGR and to quantify the packing of residues. This is important to understanding of protein stability and drug design. Comparison of the accessible surface area for the uncomplexed protein and that complexed with inhibitor provides a method of assessing the goodness of packing of the residue in a protein structure or its importance in the binding of ligands [41]. If a residue loses more than 10 Å^2^ of accessible surface area during transformation from the uncomplexed to the complexed state it is considered to be very actively involved in the interaction [42]. Changes in the accessible surface area of all residues involved in the binding of compounds within the active site of GCGR were calculated using NACCESS.

**Figure 5:**
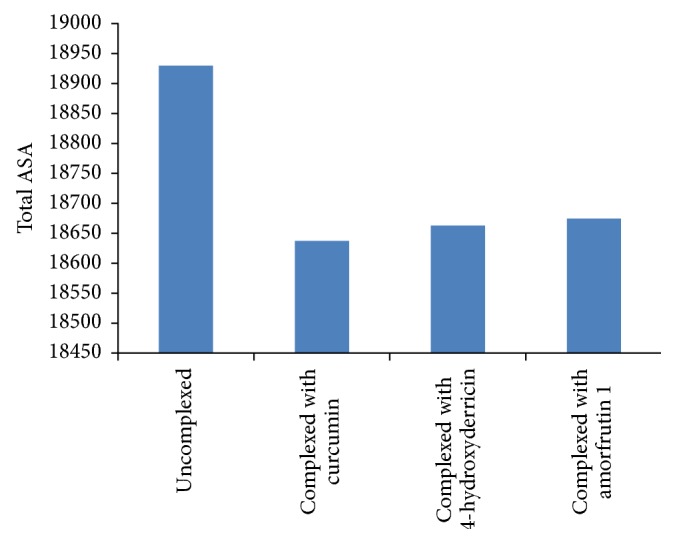
Change in total ASA of GCGR (uncomplexed and complexed).

Changes in accessible surface area (ΔASA) in Å^2^ of the interacting residues of GCGR (uncomplexed) and in complex with curcumin, 4-hydroxyderricin, and amorfrutin 1 are shown in Tables 2 and 3. Changes in the total accessible surface area of GCGR before and after its interaction with the selected compounds were also calculated (Table 2). The results revealed that the uncomplexed GCGR had a total ASA of 18,930.488 Å^2^, which was reduced to 18,637.703, 18,661.982, and 18,674.038 Å^2^ after its complex formation with curcumin, 4-hydroxyderricin, and amorfrutin 1, respectively. This large change in the accessible surface area of GCGR provides solid evidence of the effectiveness of these selected compounds. Changes in the accessible surface area (ASA) in response to complex formation for each amino acid are shown in Table 3 and Figure 5. Many residues were found to have more than 10 Å^2^ of accessible surface area complex formation. For example, M231 had an ASA of 32.642 Å^2^, which decreased to 9.531, 0.767, and 9.363 Å^2^ after the binding of curcumin, 4-hydroxyderricin, and amorfrutin 1, respectively. Similar results were observed in the case of other active site residues (L307, V311, E362, V363, F365, and L386), which undergo a high reduction in ASA before and after binding of the selected natural compounds. This encompasses the small, standard, and large interface sizes as discussed by Conte et al. [43] and thus represents a good sampling of the space of protein interfaces.

## 4. Conclusion

This study explored molecular interactions between GCGR and some well-known antidiabetic natural compounds. Molecular docking studies and their reevaluation using the *x*-score suggest that curcumin, amorfrutin 1, and 4-hydroxyderricin have higher scores than other natural compounds. The large change in the accessible surface area of the amino acid residues involved in the interaction also explains the efficacy of the binding of these compounds. Analysis of ASA further explores the important active site amino acid residues. Such information may also aid in future design of versatile GCGR-inhibitors. Overall, identification of these natural compounds may lead to design of a potent drug to combat type 2 diabetes with minimal side effects.

## Supplementary Material

Supplementary data: Schematic representation of the in silico protocol used in this study.

## Figures and Tables

**Table 1:** Residues involved in binding all the finally selected compounds against GCGR.

Compounds	Gold fitness score	*x*-score (Kcal/mol)	Residues involved	
			Hydrogen bonding	Hydrophobic interaction
Curcumin	48.53	−8.35	Y149, I235	Y145, K187, V191, I194, D195, M231, I235, E362, F365, L386
Amorfrutin 1	42.18	−8.37	K187	M231, Q232, I235, Y239, L307, V311, E362, V363, F365
4-hydroxyderricin	39.06	−8.56	No H-bond	Y145, K187, V191, I194, M231, I235, E362, F365, L386

**Table 2:** Total change in ASA of GCGR in uncomplexed and complexed form.

Complexed/uncomplexed	Change in ASA (Å^2^)
4L6R (UC)	18930.488
4L6R (complexed with curcumin)	18637.703
4L6R (complexed with 4-hydroxyderricin)	18661.982
4L6R (complexed with amorfrutin 1)	18674.038

**Table 3:** Change is ASA (Å^2^) of important active site residues of GCGR.

Complexed/uncomplexed	Y145	K187	V191	I194	M231	Q232	I235	L307	V311	E362	V363	F365	L386
4L6R (UC)	32.032	16.624	14.878	11.591	32.642	40.005	25.348	25.793	32.228	35.789	45.668	82.247	48.372
4L6R (complexed with curcumin)	4.39	2.329	0	0.439	9.531	35.199	0.574	11.298	12.427	3.194	45.668	44.311	5.973
4L6R (complexed with 4-hydroxyderricin)	17.71	2.547	0	0.006	0.767	18.509	0	10.332	19.146	7.45	45.668	43.348	15.966
4L6R (complexed with amorfrutin 1)	32.032	1.797	6.628	11.591	9.363	22.045	0	1.915	0.657	0.423	36.381	41.514	28.608

## Abbreviations

GCGR: Glucagon G-protein coupled receptor
GPCR: G-protein coupled receptor
T2DM: Type 2 diabetes mellitus
GOLD: Genetic Optimization for Ligand Docking
ASA: Accessible surface area
Cff: Consistent force field.
