# Supplementary figures and images for: Interaction of Glucagon G-Protein Coupled Receptor with Known Natural Antidiabetic Compounds: Multiscoring In Silico Approach
Source: Evid Based Complement Alternat Med. 2015 Jul 6;2015:497253. doi: 10.1155/2015/497253 (PMC4508340; doi:10.1155/2015/497253)

## Supplementary file

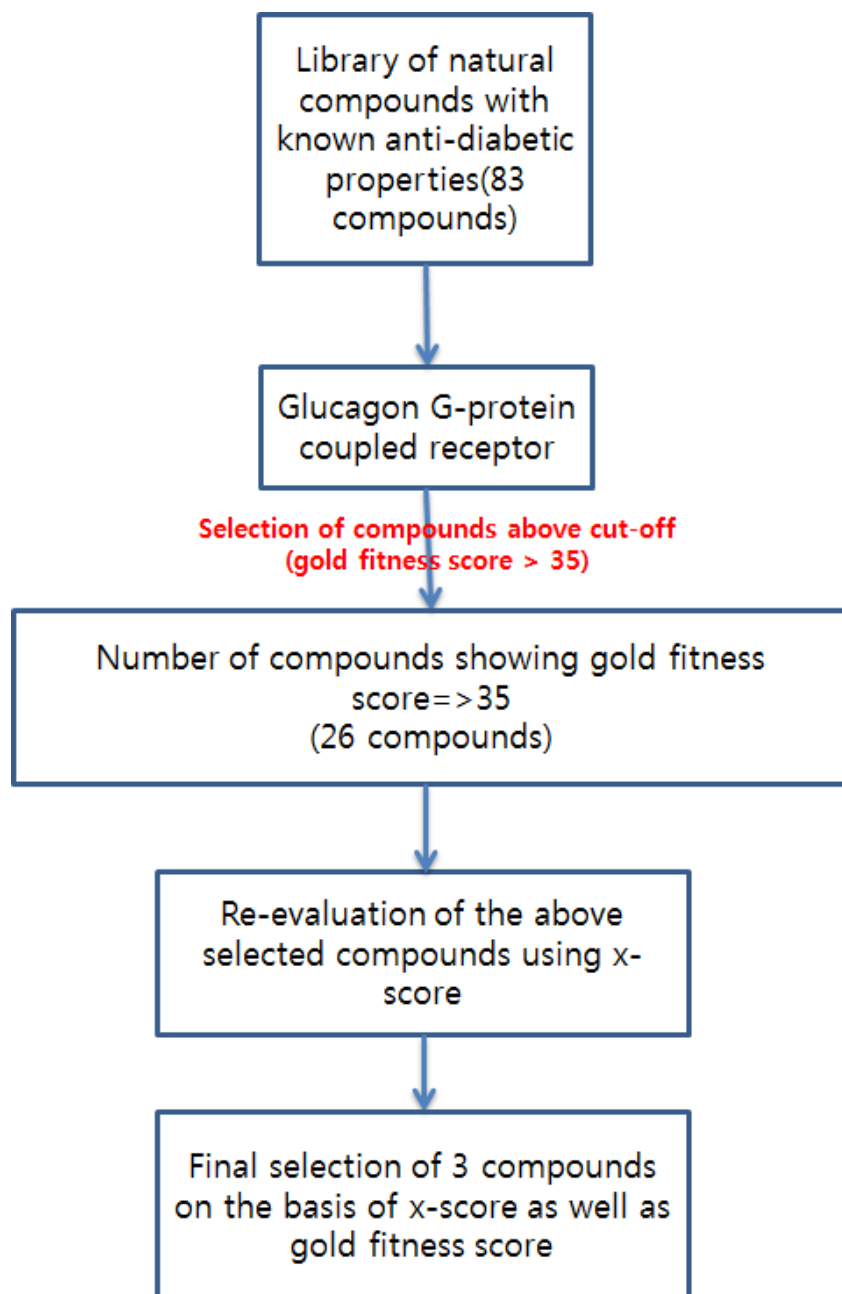

**Workflow for *in silico* protocol used in this study**

Supplement: Supplementary file 1 — Supplementary data: Schematic representation of the in silico protocol used in this study. [file 497253.f1.pdf]
